# Biomaterial-driven regenerative drug delivery: a vicennial bibliometric landscape

**DOI:** 10.3389/fmed.2025.1593985

**Published:** 2025-07-14

**Authors:** Xin Shen, Haobin Deng, Jing Lin, Jianhua Wang, Yilun Liu, Shikun Mo

**Affiliations:** ^1^Department of TCM Pharmacy, Chengdu Integrated TCM and Western Medicine Hospital, Chengdu, China; ^2^Department of Oncology, Liuzhou People’s Hospital Affiliated to Guangxi Medical University, Liuzhou, China; ^3^Department of Pharmacy, Chongzhou People’s Hospital, Chengdu, China; ^4^National Key Laboratory of Wireless Communications, University of Electronic Science and Technology of China, Chengdu, China; ^5^Surgery Ward I, Yizhou District TCM Hospital, Guangxi, China

**Keywords:** regenerative medicine, drug delivery, biomaterials, biblio metrics, tissue engeneering

## Abstract

**Introduction:**

Biomaterials are increasingly central to innovations in drug delivery and regenerative medicine, driving new therapeutic strategies for chronic diseases and tissue repair. Despite rapid growth, a comprehensive, data-driven overview of this evolving interdisciplinary landscape has been lacking.

**Methods:**

Here, we present a bibliometric analysis of articles and reviews published in the Science Citation Index Expanded (Web of Science Core Collection) from January 2005 to December 2024.

**Results and discussion:**

The findings present a bibliometric visualization that highlights key trends in publication volume, geographical distribution of research, leading institutions, top journals, research categories, and emerging keywords. Cross-disciplinary integration of biomaterials, regenerative medicine, and drug delivery is accelerating advances in stem cell-based therapies, tissue engineering, and precision drug delivery platforms. Promising frontiers include personalized medicine, organoids, organ-on-chip technologies, and digital modelling of cellular systems. However, significant challenges remain in scalability, safety, and regulatory translation. This work provides a comprehensive reference for navigating current trends and identifying future opportunities in biomaterial-driven regenerative drug delivery.

## 1 Introduction

Biomaterials are engineered to interact with biological systems for therapeutic purposes, spanning synthetic polymers to decellularized matrices designed to operate at nano- to millimetre scales (1–3). Biomaterial science investigates material—tissue interactions, while biomaterial engineering focuses on designing functional materials for clinical applications (4).

Regenerative medicine, while often conflated with tissue engineering, distinctively pursues the restoration of compromised physiological functions through integrated biological, material, and engineering strategies (5). Contemporary drug delivery systems bridge these domains by enabling spatiotemporal control over therapeutic agent release. The 1980s witnessed a pivotal milestone with the FDA approval of poly(lactic-co-glycolic acid) (PLGA)-based drug carriers, establishing biomaterials as keystones of modern controlled-release platforms (6). Subsequent paradigm shifts emerged through initiatives like the NIH Regenerative Medicine Innovation Project and EU Horizon 2020 programs. According to industry analysis, the global regenerative medicine market surpassed USD 15 billion in 2022, with annual R&D investments reaching the billions of dollars (7). Current frontiers employ smart biomaterial constructs—thermoresponsive hydrogels for minimally invasive tissue regeneration, electrospun nanofibrous scaffolds imbued with growth factors, and 3D-bioprinted organoids with embedded drug reservoirs (8–10). However, translational challenges persist: fewer than 10% of preclinical biomaterial-based delivery systems progress to Phase III trials (11). This dichotomy underscores the field’s exponential growth—publications involving “regenerative drug delivery” surged 400% (2004-2015)—against enduring barriers to clinical implementation (12).

Given the dynamic evolution of biomaterial-driven regenerative drug delivery research, implementing systematic and rigorous examination of scholarly literature emerges as an essential endeavor to refine our comprehension of the discipline’s evolving knowledge architecture. While an extensive body of articles has documented technological breakthroughs, translational barriers, and marketization trajectories within the field, such syntheses frequently demonstrate limitations in empirical methodology—particularly the overreliance on qualitative assessments rather than quantitative, data-driven evidence. This methodological gap potentially introduces variability and interpretive biases, thereby compromising systematic identification of established paradigms, core thematic priorities, and emerging frontiers. The imperative for methodological innovation in landscape analysis has been further amplified by contemporary global health exigencies, necessitating timely recalibration of conceptual frameworks to reflect post-pandemic scientific realities.

Integrating multiple analytical dimensions, this study conducted a structural bibliometric analysis to holistically map the evolving research landscape of biomaterial-driven regenerative drug delivery over the past two decades. Through systematic examination of publication distribution patterns, international collaboration networks, institutional contribution metrics, thematic progression pathways, interdisciplinary integration trends, and dissemination patterns in core journals, this investigation provides a multidimensional reconstruction of the field’s developmental framework. This framework will not only facilitate researchers across different disciplines in navigating the vast landscape of the field but also provide a valuable reference for newcomers, helping them identify promising research directions and gain deeper insights into emerging trends.

## 2 Results

### 2.1 Scientific output

**FIGURE 1:**
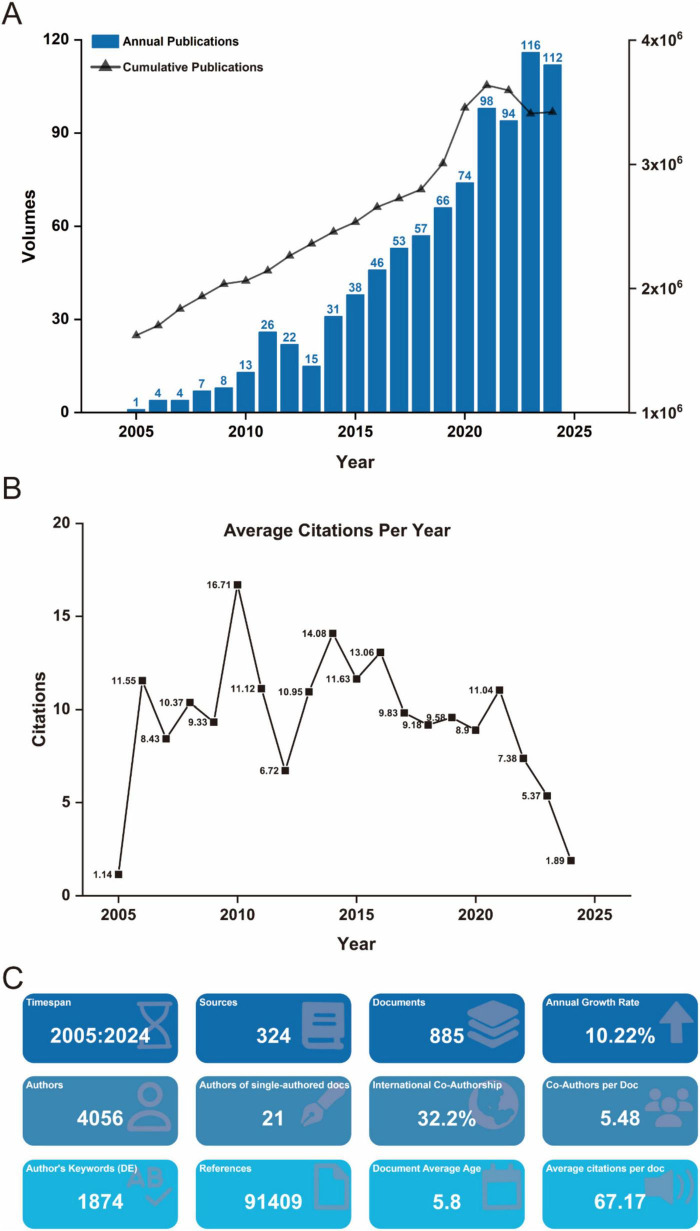
**(A)** Annual and cumulative output of global biomaterial-driven regenerative drug delivery research publications from 2005 to 2024. **(B)** Average citations per publication per year from 2005 to 2024. **(C)** Additional statistics on biomaterial-driven regenerative drug delivery publications from R bibliometrix.

Between January 1, 2005, and December 31, 2024, a total of 885 scholarly publications on biomaterial-driven regenerative drug delivery were recorded globally (Figure 1A). Annual publication output exceeded 50 in 2017, and rose above 100 in 2024, peaking at 116 in 2023. This sharp increase suggests that the COVID-19 pandemic substantially accelerated research activity in this field. The publication rate exhibited a max annual growth of 32.4%, with annual output from 2005 to 2024. Citation analysis indicates an average of 9.41 citations per publication per year, with the highest impact observed in 2010 at 16.71 citations per year (Figure 1B). Further insights into journal distribution, citation patterns, and bibliometric trends are provided in Figure 1C.

### 2.2 Countries/regions

**FIGURE 2:**
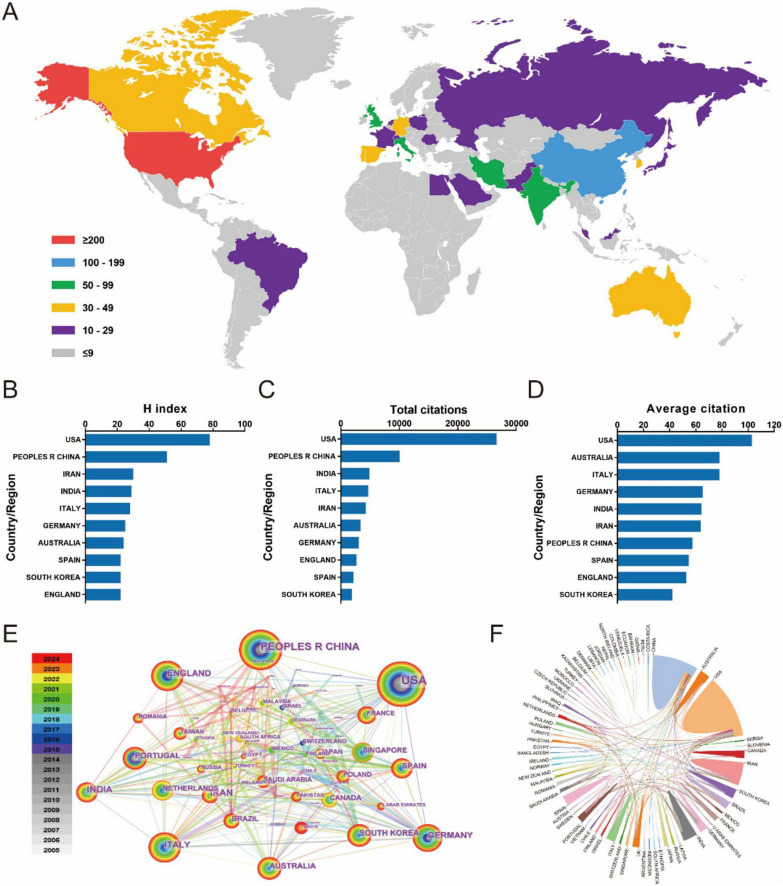
**(A)** The distribution of biomaterial-driven regenerative drug delivery from 2005 to 2024. **(B–D)** H index, total citations, and average citation. **(E)** Country/region network visualization produced across 77 countries/regions worldwide. **(F)** Country/region crosschord charts.

Seventy seven countries/regions contributed to global scientific productivity, with geographic contributions visualized through publication density mapping (Figure 2A). Table 1 lists the top 10 countries/regions with the most published articles. The USA led research output (259 publications), followed by China (175) and India (76). When measuring research impact through H-index metrics, the USA maintained dominance (*H* = 78), with China (*H* = 51), and Iran (*H* = 30) completing the top tier (Figure 2B). Citation analysis revealed the USA as the primary knowledge disseminator, with China and India emerging as secondary contributors in total citations. Notably, Australia and Italy demonstrated exceptional performance in average citation rates, ranking second and third respectively (Figures 2C, D). Network analysis identified the United States and China as central nodes in global research collaboration, with Germany, United Kingdom, France, India, Italy, South Korea, and Australia comprising additional key contributors (Figure 2E). The collaboration network exhibits extensive globalization patterns, featuring three principal knowledge exchange hubs: North America, Europe, and East Asia (Figure 2F). This tripartite structure underscores the intercontinental nature of contemporary scientific cooperation.

**TABLE 1:** Top 10 productive countries/regions in biomaterial-driven regenerative drug delivery, ranked by the number of publications.

Rank	Country/Region	Article counts	Percentage of total (%, N/885)
1	United States	259	29.266
2	China	175	19.774
3	India	76	8.588
4	Iran	67	7.571
5	Italy	60	6.78
6	England	51	5.763
7	Germany	47	5.311
8	South Korea	45	5.085
9	Australia	43	4.859
10	Spain	40	4.52

### 2.3 Institutions

**FIGURE 3:**
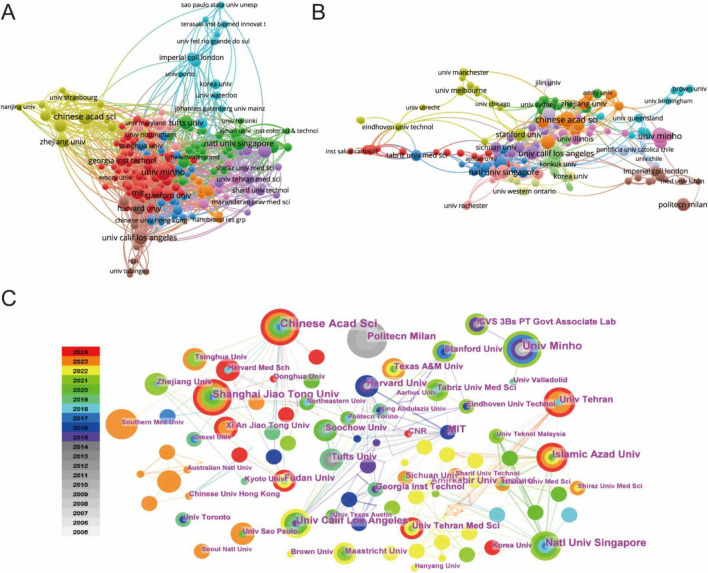
**(A)** Analysis of country/regional collaboration using Vosviewer. **(B)** Analysis of institutional collaboration using Vosviewer. **(C)** Institution network visualization produced using CiteSpace.

The global research landscape encompasses over 1,300 participating institutions, as visualized through comprehensive institutional mapping (Figure 3). Publication leaders are detailed in Table 2, where Harvard University and the University of California System emerge as the most productive institutions (26 publications each), followed closely by the Chinese Academy of Sciences (24 publications) and Universidade do Minho (20 publications). Bibliometric analysis utilizing VOSviewer software revealed distinct dimensions of scientific influence (Figures 3A, B). Collaborative network mapping (Figure 3A) identifies three predominant knowledge hubs centered around the Chinese Academy of Sciences, Zhejiang University, and the National University of Singapore. Citation network analysis (Figure 3B) demonstrates dense interconnection patterns among high-output institutions, suggesting reciprocal citation behaviors that reinforce their disciplinary leadership. Temporal network visualization tracks the progressive expansion of research contributions, with China, the USA, and European nations driving biomaterial-focused advances in regenerative drug delivery systems (Figure 3C).

**TABLE 2:** Top 10 institutions published literature related to the field of biomaterial-driven regenerative drug delivery, ranked by the number of publications.

Rank	Institution	Article counts	Percentage of total (%, N/885)	Country
1	Harvard University	26	2.938	USA
2	University of California System	26	2.938	USA
3	Chinese Academy of Sciences	24	2.712	China
4	Universidade do Minho	20	2.26	Portugal
5	National University of Singapore	17	1.921	Singapore
6	Ciber Centro de Investigacion Biomedica en Red	16	1.808	Spain
7	CIBER-BBN	16	1.808	Spain
8	University of California Los Angeles	16	1.808	USA
9	Tufts University	15	1.695	USA
10	Massachusetts Institute of Technology (MIT)	14	1.582	USA

**FIGURE 4:**
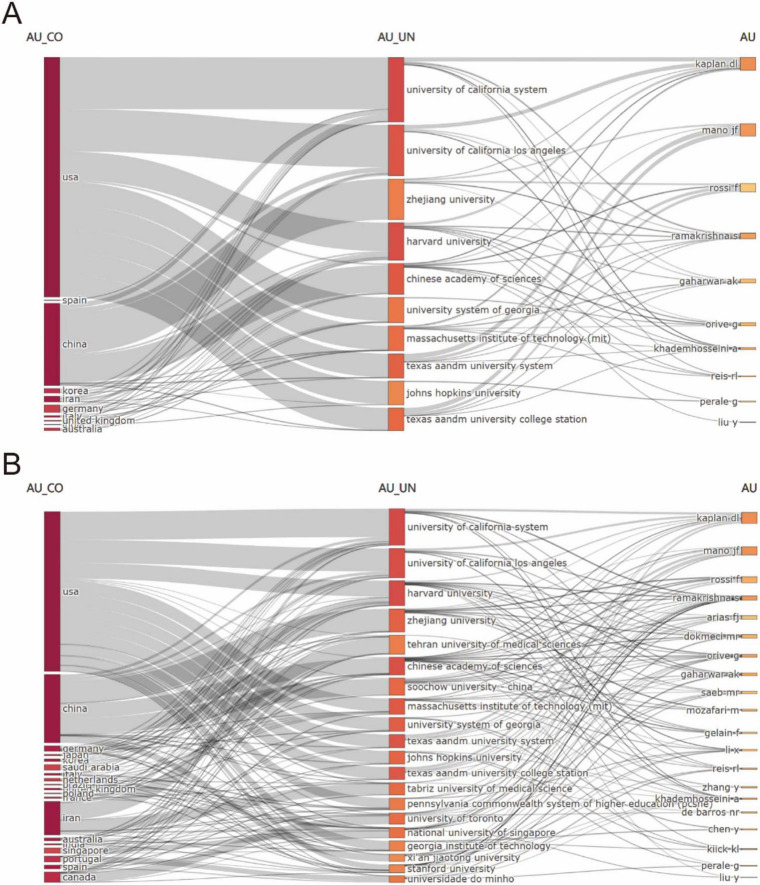
**(A)** Interconnections of the top 10 high-productivity countries/regions, institutions, and authors.**(B)** Interconnections of the top 20 high-productivity countries/regions, institutions. and authors.

Figure 4 illustrated the global research landscape in biomaterial-driven regenerative drug delivery, mapping country-level contributions, institutional affiliations, and key authors. Figure 4A highlighted the dominance of the USA and China, with leading institutions such as the University of California System, Zhejiang University, and the Chinese Academy of Sciences playing pivotal roles. Prominent researchers, including Kaplan DL, Mano JF, and Ramakrishna S, were linked to these institutions. Figure 4B expanded the scope, showcasing a broader international network, with new contributors from Japan, Saudi Arabia, and Canada, as well as additional universities such as Tehran University of Medical Sciences and Soochow University. Collectively, these multi-dimensional visualizations confirmed two critical trends: the formation of self-reinforcing academic ecosystems among top-tier institutions, and the increasing globalization of interdisciplinary research in advanced therapeutic development. The convergence of spatial network density and temporal expansion metrics underscored the field’s transition toward increasingly complex, internationally integrated research paradigms.

### 2.4 Journals and research area

**FIGURE 5:**
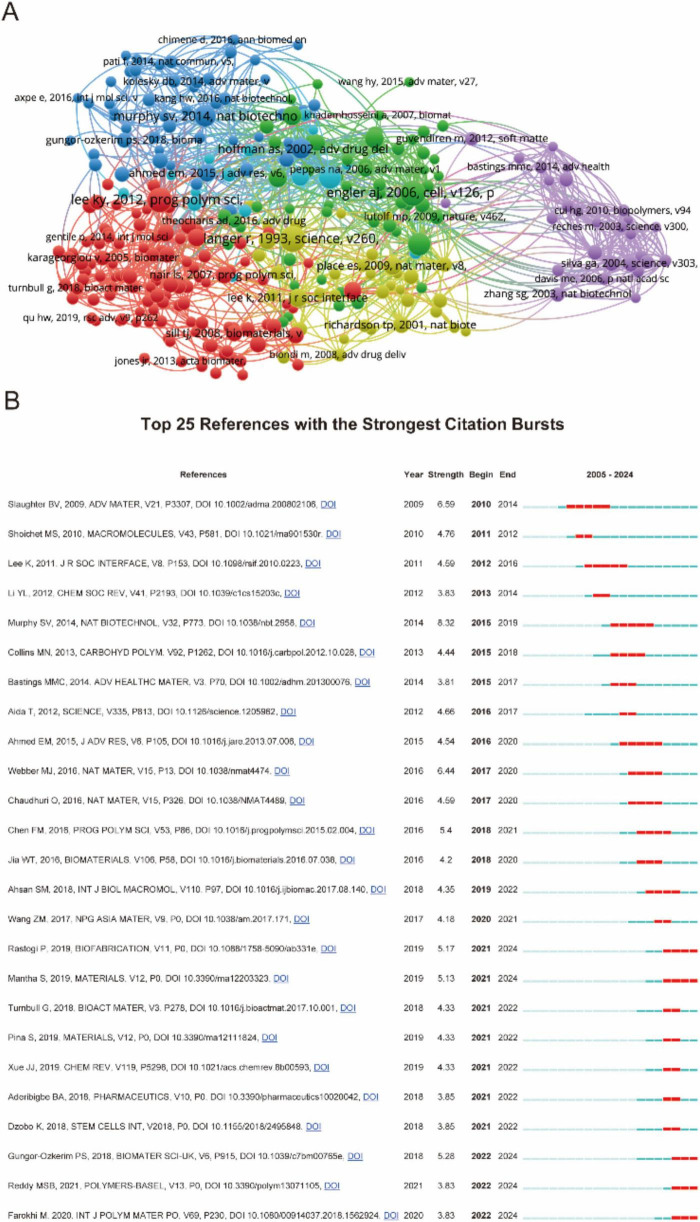
**(A)** Top 10 journals in biomaterial-driven regenerative drug delivery based on publication counts. **(B)** Journal output trends within the top 10 from 2005 to 2024. **(C)** Delineation of core and non-core journals according to Bradford’s law.

The analysis encompassed 324 journals contributing to global research dissemination, as visualized in Figure 1C. Figure 5A presents the decadal ranking of publication productivity, with ACTA Biomaterialia and Biomaterials emerging as the predominant sources in this domain. The remaining high-yield journals primarily concentrated on biomaterial engineering, molecular sciences, and pharmaceutical delivery systems. Temporal distribution patterns revealed in Figure 5B demonstrate consistent expansion of scholarly output, particularly showing accelerated progression post-2015. Application of Bradford’s law of scattering facilitated the stratification of journals into three distinct zones (Figure 5C; Tables 3, 4), identifying 19 core dissemination channels within Zone 1 that accounted for the majority of impactful publications. Table 5 revealed that the dominant research domains identified using VOSviewer include Materials Science, Engineering, Chemistry, Polymer Science, and Science Technology Other Topics. These focal areas underscored the primary directions of current research and point to promising avenues for future advancements in the field.

**TABLE 3:** Nineteen core source journals in zone 1 according to Bradford’s law.

Rank	Journal	Article counts
1	Acta Biomaterialia	33
2	Biomaterials	31
3	Polymers	23
4	International Journal of Molecular Sciences	19
5	Advanced Healthcare Materials	18
6	International Journal of Biological Macromolecules	18
7	Advanced Drug Delivery Reviews	16
8	Materials	16
9	Frontiers in Bioengineering and Biotechnology	15
10	ACS Biomaterials Science & Engineering	13
11	Advanced Functional Materials	13
12	Pharmaceutics	13
13	Gels	12
14	Biomacromolecules	11
15	Journal of Biomedical Materials Research Part A	11
16	Biomaterials Science	10
17	Molecules	10
18	ACS Applied Bio Materials	9
19	Carbohydrate Polymers	9

**TABLE 4:** According to Bradford’s law, the 632 journals were classified into zones 1–3.

Zone	No. of journals	No. of publications	Percentage
1	19	300	33.89%
2	67	294	33.22%
3	238	291	32.89%
Total	324	885	100%

**TABLE 5:** Top 10 focused research areas.

Rank	Research areas	Records	Percentage of total (%, N/287)
1	Materials Science	389	43.955
2	Engineering	236	26.667
3	Chemistry	216	24.407
4	Polymer science	150	16.949
5	Science technology other topics	124	14.011
6	Pharmacology Pharmacy	107	12.09
7	Biochemistry Molecular Biology	104	11.751
8	Physics	69	7.797
9	Biotechnology Applied Microbiology	68	7.684
10	Cell Biology	58	6.554

### 2.5 References and funds

**FIGURE 6:**
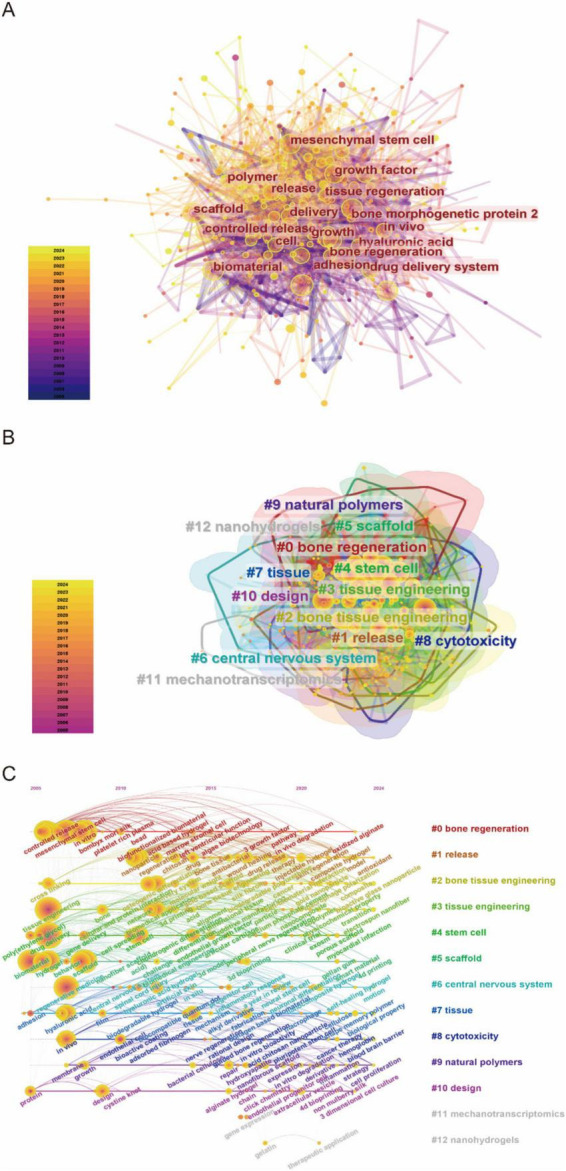
**(A)** Mapping of references based on Vosviewer from 2005 to 2024. **(B)** Top 35 references with the strongest citation bursts.

Figure 6 presented a comprehensive citation analysis of influential references in biomaterial-driven regenerative drug delivery. Figure 6A illustrated a network of highly cited papers, grouped into thematic clusters such as biomaterials, bioengineering, drug delivery, and regenerative medicine. Figure 6B ranked the top 25 references with the strongest citation bursts. The recent citation bursts (2021–2024) indicated emerging research directions, particularly in bioactive materials and hydrogels for biomedical applications. Together, these analyzes provided valuable insights into the evolution and impact of foundational research in biomaterial-driven regenerative drug delivery.

Table 6 provided an overview of the funding sources supporting research on biomaterial-driven regenerative drug delivery studies. In the table, United States Department of Health and Human Services (HHS) stood out as the primary funders, supporting a total of 124 articles. This was closely followed by National Institutes of Health (NIH) USA with 123 articles, National Natural Science Foundation of China (NSFC) with 95 articles, and National Science Foundation (NSF) with 72 articles. Interestingly, the allocation of funding closely reflected the results of the country-based analysis, emphasizing the significant contributions of the United States and China as key drivers of research in this field.

**TABLE 6:** Top 10 funds related to the field of biomaterial-driven regenerative drug delivery from 2005 to 2024.

Rank	Agencies	Article counts	Percentage
1	United States Department of Health and Human Services (HHS)	124	14.011
2	National Institutes of Health (NIH) USA	123	13.898
3	National Natural Science Foundation of China (NSFC)	95	10.734
4	National Science Foundation (NSF)	72	8.136
5	European Union (EU)	38	4.294
6	Fundacao para a Ciencia e a Tecnologia (FCT)	25	2.825
7	Spanish Government	24	2.712
8	National Research Foundation of Korea	23	2.599
9	European Research Council (ERC)	20	2.26
10	Natural Sciences and Engineering Research Council of Canada (NSERC)	20	2.26

### 2.6 Keyword visualization and burst test

**FIGURE 7:**
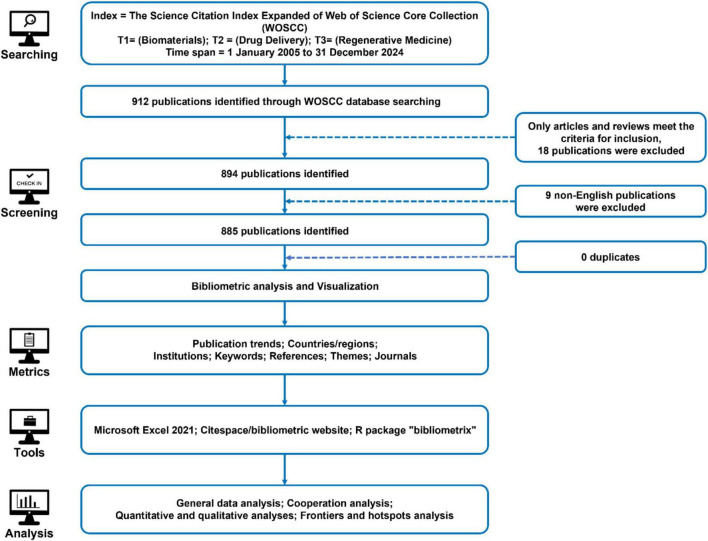
**(A)** Keyword network visualization produced using CiteSpace. **(B)** Cluster information for keywords. **(C)** Keyword timeline visualization map for biomaterial-driven regenerative drug delivery.

Recent advancements in tissue engineering and biomaterials research demonstrated marked thematic evolution and interdisciplinary convergence. Network analysis in Figure 7A illustrated a robust interconnection of research domains, notably mesenchymal stem cells, scaffold architectures, and bone regeneration. Temporal color-coding revealed intensifying focus on controlled release platforms and regenerative biomaterials in recent years. Clustering analysis (Figure 7B) identified natural polymers (Cluster #9), stem cell therapies (#4), and bone tissue engineering (#2) as dominant thematic nuclei. The distinct emergence of nanohydrogels (Cluster #12) underscored nanotechnology’s rising influence, reflecting a paradigm shift toward multifunctional biomaterials with enhanced regenerative precision. Temporal keyword progression in Figure 7C highlighted the ascendance of bone regeneration (Cluster #0) and release kinetics (#1) as central research themes. A chronochromatic gradient (2010–2020) captured dynamic prioritization shifts, revealing an accelerating integration of nanoscale engineering and optimized tissue scaffolding frameworks.

**FIGURE 8:**
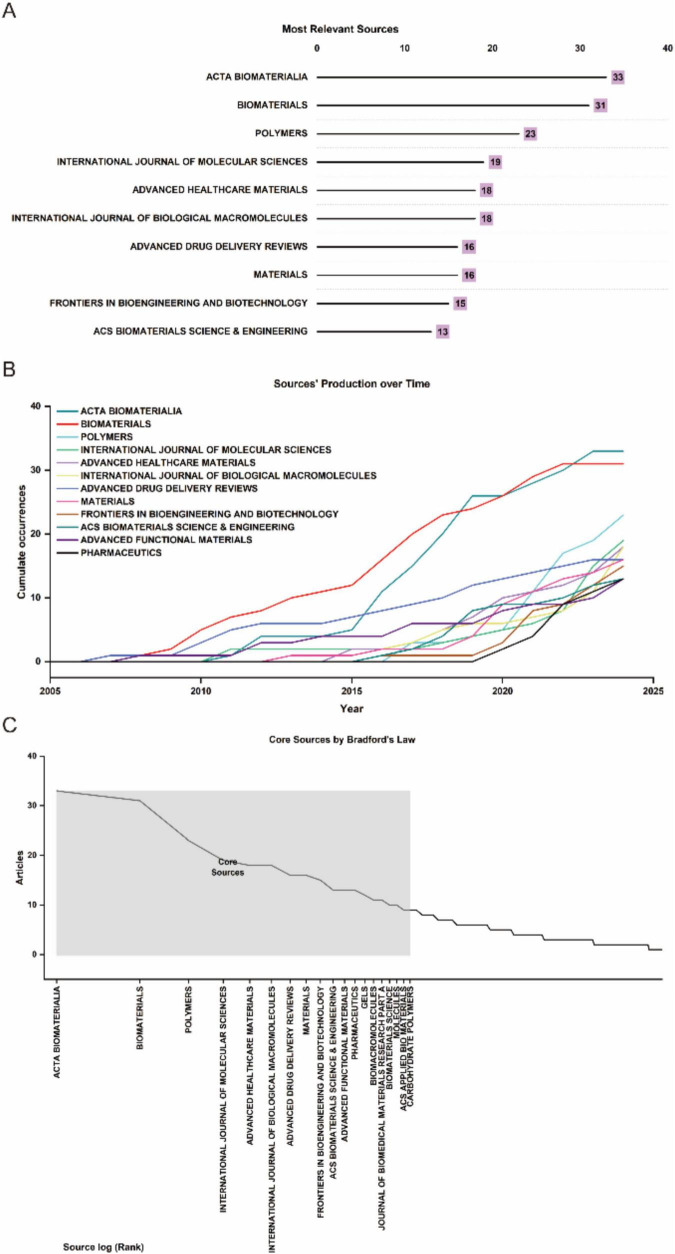
Top 15 keywords with the strongest citation bursts, arranged in ascending order based on burst time.

Burst detection analysis identified keywords demonstrating rapid frequency surges over discrete time intervals, revealing critical research hotspots and emerging research fronts. Figure 8 presented the top 15 keywords ranked by burst strength magnitude.

## 3 Discussion

### 3.1 Analysis of the geographical distribution of publications

The global distribution of biomaterial-driven regenerative drug delivery research illustrates a robust and expanding international interest in this transformative field. As depicted in Figure 1A, the annual and cumulative publication outputs have demonstrated a consistent increase from 2005 through 2024, reflecting not only growing scientific interest but also substantial progress in the field’s evolution. The rising number of publications highlights a clear global commitment to advancing regenerative medicine, with substantial contributions from various research hubs across the world.

North America and Europe have historically been at the forefront of this research, with the United States leading the charge. As shown in Table 1, the USA accounts for nearly 30% of the total publications, reinforcing its dominant role in driving innovation in this space. This dominance is further reflected in Figure 2B, which ranks the USA at the top in terms of the H-index, a metric indicative of both productivity and citation impact. Meanwhile, China has emerged as a significant player in the field, ranking second in terms of total publications, while India, Iran, and Italy follow closely behind, underscoring the broad global participation in advancing regenerative drug delivery technologies. However, the citation impact of these publications reveals a nuanced geographical distribution. As shown in Figure 2D, while the USA leads in overall publication volume and citation count, countries such as Australia and Italy outperform in terms of average citations per paper. This discrepancy suggests a shift toward more highly specialized, impactful research outputs from these regions. Australia, in particular, stands out for its relatively higher citation rates, likely due to the country’s focus on applied research in regenerative biomaterials. Such differences in citation impact highlight the varying research emphases across regions, from foundational studies in North America to applied, clinical translational research in Europe and Oceania.

The international collaboration network further emphasizes the interconnectedness of global research in regenerative drug delivery. Figure 3E presents a compelling visualization of how countries and regions collaborate in advancing the field. Strong collaborative ties are evident between the USA, China, and numerous European nations, fostering cross-border exchanges of ideas, data, and resources. This network of international cooperation is not only vital for accelerating scientific progress but also for ensuring the integration of diverse perspectives in addressing the complex challenges in regenerative medicine.

At the institutional level, institutions in the USA, such as Harvard University and the University of California, continue to lead in both publication output and collaboration, as seen in Figures 4A, B. These institutions not only contribute to a significant proportion of the global research but also foster institutional collaborations across borders, further enhancing the reach and impact of their work.

In conclusion, the geographical distribution of publications in biomaterial-driven regenerative drug delivery underscores a dynamic and highly collaborative global research ecosystem. While the USA, China, and Europe dominate in terms of publication volume, countries like Australia and Italy stand out for their citation impact, reflecting the regional diversity in research priorities. The expanding network of international collaborations plays a crucial role in advancing the field, fostering a global approach to solving the challenges of regenerative medicine. As this field continues to evolve, it will be shaped by the collective efforts of researchers worldwide, driven by a shared commitment to improving human health through innovative biomaterials and drug delivery technologies.

### 3.2 Analysis of journals, and studies

The dissemination of research in biomaterial-driven regenerative drug delivery is characterized by a concentration of high-impact journals, reflecting the multidisciplinary nature and growing prominence of this field. As illustrated in Figure 5A, Acta Biomaterialia and Biomaterials are the most prolific sources, serving as primary platforms for cutting-edge studies on biomaterials and regenerative therapies. Other key journals, including Polymers, Advanced Healthcare Materials, and Advanced Drug Delivery Reviews, highlight the field’s strong intersection with polymer science, nanomedicine, and pharmaceutical sciences. The longitudinal trends in journal output (Figure 5B) indicate sustained growth across the top publishing venues, with particularly sharp increases observed in Acta Biomaterialia and International Journal of Molecular Sciences. This trend underscores the expanding scope of biomaterial applications, from fundamental material design to translational drug delivery strategies. The classification of journals according to Bradford’s Law (Figure 5C; Table 3) further delineates the structure of knowledge dissemination in this field, with 19 core journals accounting for the majority of published research. This concentration of high-impact studies within a select group of sources reflects the establishment of a strong and specialized research community.

The intellectual structure of this research domain is further mapped through citation network analyses (Figure 6A), revealing dense interconnections among key studies. The presence of multiple citation bursts (Figure 6B) highlights landmark publications that have significantly shaped the field, with surges in citations corresponding to major breakthroughs in biomaterials, drug release kinetics, and tissue engineering. The temporal distribution of these bursts suggests a sustained influx of transformative contributions, reflecting the field’s continuous evolution and responsiveness to emerging biomedical challenges.

Funding support plays a critical role in steering research directions, as shown in Table 6. The United States Department of Health and Human Services (HHS) and the National Institutes of Health (NIH, United States) are the predominant funding bodies, underscoring the strategic investment in regenerative medicine within the U.S. Meanwhile, the National Natural Science Foundation of China (NSFC) and the European Research Council (ERC) contribute significantly to global research efforts, reinforcing the international scope of advancements in biomaterial-driven drug delivery.

In summary, the analysis of journal distributions and study trends underscores the maturation of biomaterial-driven regenerative drug delivery as a distinct yet highly interdisciplinary research domain. The concentration of publications in core journals, the increasing convergence of materials science, pharmaceutical research, and bioengineering, and the sustained support from leading funding agencies all point to a rapidly evolving field poised for continued innovation. As biomaterial technologies advance toward clinical translation, these insights offer a roadmap for future research directions and emerging areas of impact in regenerative medicine and drug delivery.

### 3.3 Analysis of research hotspots

The evolving landscape of biomaterials-driven regenerative drug delivery manifests through dynamic keyword clustering patterns and temporal emergence trajectories. The keyword co-occurrence network (Figure 7A) reveals tight interconnections among core themes focusing on biomaterials, tissue engineering, and controlled drug release. Cluster decomposition analysis (Figure 7B) identifies distinct research domains, while timeline visualization (Figure 7C) demonstrates chronological shifts in research priorities. Citation burst analysis (Figure 8) highlights “hydrogel” and “gene delivery” as frontier areas attracting intensified scholarly attention. These analytical approaches collectively identify three principal research directions: hydrogel-based drug delivery, stem cell and tissue engineering integration, and gene and growth factor delivery strategies.

#### 3.3.1 Hydrogel-based drug delivery systems

Hydrogel systems have consolidated their position as fundamental delivery matrices, evidenced by prominent network centrality and citation bursts (Figures 7, 8). Their listing among the top 10 recurring keywords (Table 7) confirms current research dominance. These polymeric networks combine tunable mechanical properties with spatiotemporal drug release modulation and improved physiological compatibility, enabling applications spanning targeted therapeutics to regenerative wound management (13–15). The emergence of “injectable hydrogels” as a high-burst keyword (Figure 8) reflects growing emphasis on minimally invasive clinical implementations (16, 17). Recent innovations incorporating supramolecular architectures and nanoparticle-based hydrogel composites demonstrate expanding potential for precision medicine applications. DeFrates et al. (18) proposed that supramolecular hydrogels formed by self-assembly of PEG-DPCA conjugates could enable sustained delivery of DPCA for the palliation of inflammatory bowel disease (IBD). In addition, Xiong et al. (19) designed a novel regeneration-directing artificial skin (RDAS) system, which is based on the rational design of multilayered hydrogel and can approximate the natural skin matrix. The RDAS offers a cell-free antiscarring therapeutic strategy for regenerative wound healing, resulting in improved outcomes.

**TABLE 7:** The top 10 keywords with the highest frequency in biomaterial-driven regenerative drug delivery.

Rank	Keyword	Frequency	Year
1	Drug delivery	446	2006
2	Regenerative medicine	300	2007
3	Tissue engineering	277	2006
4	*In vitro*	193	2008
5	Biomaterial	159	2005
6	Mesenchymal stem cell	151	2007
7	Scaffold	141	2008
8	Stem cell	122	2011
9	Hydrogel	111	2006
10	Controlled release	101	2006

#### 3.3.2 Stem cell and tissue engineering integration

Integration of stem cell therapies with biomaterial scaffolds constitutes a persistent research focus, as indicated by sustained prominence of “mesenchymal stem cells” and “tissue engineering” in keyword rankings (Table 7). Network topology analysis (Figure 7A) positions mesenchymal stem cells at the intersection of biomaterial and scaffold development discussions, underpinning their crucial role in directing tissue restoration processes (20, 21). Temporal keyword evolution (Figure 7C) documents progressive specialization in scaffold-mediated cell deployment systems, paralleled by material science advancements enabling precise regulation of stem cell differentiation within engineered constructs (22, 23). This materials-biology synergy propels development toward patient-specific regenerative solutions.

#### 3.3.3 Gene and growth factor delivery strategies

Emerging citation bursts for “gene delivery” and “growth factor delivery” (Figure 8) signal intensified investigation into molecular therapeutic enhancement. These strategies seek to amplify biomaterial performance through controlled mobilization of cellular recruitment and differentiation mechanisms. The recurrent “controlled release” terminology (Table 7) reflects persistent optimization efforts for spatial-temporal regulation of bioactive payloads—balancing sustained therapeutic action with reduced systemic exposure (24, 25). Klipp et al. (26) introduced a calcium-responsive system designed to enhance endosomal escape through non-covalent capturing of PLC to the TFAMoplex followed by its release within endosomes and nanobody-mediated targeting to the endosomal membrane, which offers the prospect of improved delivery of macromolecules, especially nucleic acids. Moreover, Li et al. (27) developed a spatiotemporally controllable microneedle (MN) drug delivery platform that delivers the methoxy polyethylene glycol-polyethyleneimine (mPEG-PEI) modified metal-organic frameworks (MOFs) sonosensitizer and the clustered regularly interspaced short palindromic repeats-activating (CRISPRa)/CRISPRa-uncoupling protein 1 (UCP1) system intradermally to adipocytes. And this therapy platform is capable of achieving two major strategies of “annihilation” and “countermeasure”: one is to kill redundant white adipocytes by sonodynamic therapy, and the other is to promote the browning of white adipocytes through the controllable release of CRISPRa-UCP1 system and sonodynamic effect.

### 3.4 Future research trends

The future of biomaterial-driven regenerative drug delivery is set to be defined by a convergence of biomaterials science, tissue engineering, and precision medicine. Insights from keyword networks (Figure 7A), cluster analyses (Figure 7B), and citation burst data (Figure 8) reveal a rapidly evolving research landscape, with emerging themes that will likely shape the next phase of innovation. Three key research trajectories can be anticipated: smart and responsive biomaterials, advanced cell- and gene-based therapies, and personalized, precision drug delivery systems. These trends highlight a shift toward more adaptive, biologically integrated, and patient-specific therapeutic strategies.

#### 3.4.1 Smart and responsive biomaterials

A major direction in future biomaterials research will be the development of smart and stimuli-responsive materials capable of dynamically interacting with their biological environment. The growing prominence of injectable hydrogels and supramolecular hydrogel systems (Figure 8) reflects an increasing demand for biomaterials that offer enhanced tunability and functionality. These next-generation materials will be engineered to respond to specific physiological stimuli—such as pH changes (28, 29), enzymatic activity (30, 31), or external triggers (32–34) (e.g., light, ultrasound, or magnetic fields)—enabling controlled and sustained drug release. Advances in self-healing biomaterials and nanoengineered scaffolds will further enhance their adaptability, providing a new level of precision in tissue regeneration and drug delivery. Such innovations are poised to transform regenerative medicine by improving treatment efficacy and patient compliance.

#### 3.4.2 Advanced cell- and gene-based therapies

The integration of biomaterials with cell- and gene-based therapies represents another major frontier. The continued prominence of stem cell and tissue engineering keywords in research networks (Figure 7) suggests that future regenerative strategies will focus on optimizing biomaterial platforms for cellular encapsulation, differentiation, and *in situ* tissue regeneration. In parallel, the strong citation bursts for gene delivery (Figure 8) indicate a growing emphasis on combining biomaterials with genetic engineering approaches, including CRISPR-based therapies (35, 36) and RNA therapeutics (37, 38). Future research will prioritize the development of biomaterials that can precisely control the spatiotemporal release of therapeutic genes, exosomes, and growth factors—offering unprecedented control over cellular function and regeneration (39, 40). These advances hold the potential to revolutionize regenerative medicine, moving from passive scaffold-based approaches to active, instructive biomaterial systems.

#### 3.4.3 Personalized and precision drug delivery systems

Personalized medicine is expected to drive the next wave of innovation in biomaterial-based drug delivery. The sustained prominence of controlled release as a high-frequency keyword (Table 7) underscores the field’s ongoing effort to optimize pharmacokinetics and bioavailability. Future research will emphasize patient-specific biomaterial formulations, integrating machine learning and AI-driven predictive modeling to design customized drug delivery platforms (41, 42). Bioprinting technologies will play an increasing role in fabricating individualized constructs tailored to specific patient needs (43, 44). Additionally, the integration of nanotechnology with biomaterials will enable the development of multifunctional carriers for targeted drug delivery, minimizing systemic side effects while maximizing therapeutic outcomes (45, 46). These advancements will pave the way for a new era of precision regenerative therapies, where treatments are designed at the intersection of biomaterials science, computational modeling, and clinical translation.

In conclusion, as biomaterial-driven regenerative drug delivery continues to evolve, the focus is shifting toward intelligent, adaptable, and highly personalized therapeutic solutions. The development of responsive biomaterials, the integration of gene and cell-based therapies, and the emergence of precision medicine approaches will define the next decade of innovation. The challenge ahead lies in translating these cutting-edge technologies from the laboratory to clinical practice, necessitating closer collaboration between materials scientists, bioengineers, and clinicians. By bridging fundamental research with translational applications, the field is poised to redefine regenerative medicine, offering unprecedented possibilities for tissue repair, drug delivery, and patient-specific therapeutics.

### 3.5 Limitations of this study

While this study provides valuable insights into global advancements and research trends in biomaterial-driven regenerative drug delivery, certain limitations must be acknowledged. A key challenge lies in fully elucidating the complex interplay between biomaterials, drug release dynamics, and regenerative processes within physiologically relevant microenvironments. In particular, the precise molecular mechanisms by which biomaterials influence drug bioavailability, cellular signaling, and tissue remodeling, remains insufficiently understood. Addressing these knowledge gaps is critical for the rational design of biomaterials that enhance drug delivery efficacy and promote tissue regeneration.

Additionally, this study may be subject to selection biases arising from database constraints and language restrictions. The exclusion of major repositories such as PubMed, Cochrane, and Embase, along with the omission of non-English literature, may have limited the comprehensiveness of the analysis. Moreover, the reliance on citation frequency as a primary metric may undervalue high-impact yet recently published studies that have not yet accumulated extensive citations, potentially distorting bibliometric trends and underrepresenting emerging innovations in biomaterial-based drug delivery.

Future research should adopt a more integrative approach, drawing from a broader range of databases and multilingual sources to ensure a more comprehensive and inclusive analysis. Additionally, refining bibliometric methodologies to account for emerging yet under-cited studies could provide a more accurate representation of the evolving landscape in biomaterials and regenerative drug delivery. Such efforts will be instrumental in advancing the development of next-generation biomaterials with precisely engineered drug release profiles, optimized biocompatibility, and enhanced regenerative potential, ultimately driving more effective clinical applications in regenerative medicine.

## 4 Conclusion

Our research highlights significant global trends and an increasing emphasis on biomaterial-driven regenerative drug delivery from 2005 to 2024. The United States has emerged as a leading contributor, demonstrating high citation frequencies and H-index scores, underscoring its pivotal role in advancing this field. This analysis reinforces the United States’ leadership in driving innovation at the intersection of biomaterials, drug delivery, and regenerative medicine. Furthermore, our study identifies several key directions for future research, including smart and Responsive Biomaterials, advanced cell- and gene-based therapies, and personalized and precision drug delivery systems to optimize regenerative outcomes. These focal points are expected to shape the next generation of biomaterial-based therapies, unlocking novel opportunities to enhance drug efficacy, improve tissue regeneration, and address complex pathological conditions with greater precision.

## 5 Experimental section

### 5.1 Data source

The Web of Science Core Collection (WOSCC) was selected as the primary data source for this bibliometric analysis due to its comprehensive coverage and methodological reliability. Biomaterials-driven regenerative drug delivery is a highly interdisciplinary field, spanning materials science, medicine, chemistry, pharmacy, and bioengineering. A robust bibliometric study requires an integrated database that captures research across these domains to provide an accurate and representative assessment of global research trends. WOSCC offers several key advantages. First, its inclusion of cited reference data enables in-depth knowledge mapping, facilitating a deeper understanding of the interconnections between biomaterials, drug delivery, and regenerative medicine. Second, WOSCC provides citation reports that serve as validation tools, ensuring the accuracy, reliability, and reproducibility of bibliometric analyses. Third, WOSCC datasets are natively compatible with leading bibliometric software, eliminating the need for format conversion and minimizing risks of data corruption or missing fields, thereby preserving analytical integrity. Furthermore, WOSCC encompasses the Science Citation Index Expanded (SCIE), ensuring a high level of quality control by indexing rigorously vetted journals and high-impact publications. Additionally, its journal selection follows Bradford’s Law and Garfield’s Law, ensuring that core publications are effectively captured while minimizing the risk of omissions. Despite these strengths, reliance on a single database presents inherent limitations. The exclusion of other major repositories such as PubMed, Cochrane, and Embase, as well as non-English publications, may introduce selection biases. To enhance the comprehensiveness and inclusivity of future bibliometric assessments in biomaterials-driven regenerative drug delivery, integrating multiple databases and expanding linguistic coverage will be essential.

### 5.2 Retrieval strategy

Biomaterials play a pivotal role in regenerative medicine, with advanced drug delivery systems and bioengineered scaffolds driving innovation. However, bibliometric analyses in such a dynamic field require a nuanced retrieval strategy to account for the complexity of research co-occurrence dynamics. Restricting retrieval to widely recognized terms related to biomaterials and drug delivery risks overlooking emerging yet impactful innovations. Conversely, such an approach may inadvertently amplify the prominence of well-established research entities, reinforcing existing publication biases and skewing the representation of emerging contributions. To address these challenges, we implemented an analytical framework designed to balance inclusivity and specificity. This strategy ensures that both foundational and emerging trends in biomaterials-driven regenerative drug delivery are accurately captured, providing a more representative and unbiased view of the field. Our overarching goal was to map the evolving research landscape, uncovering key patterns and trajectories that make this analysis broadly relevant and accessible to scientists, clinicians, and industry leaders shaping the future of regenerative medicine.

**FIGURE 9:**
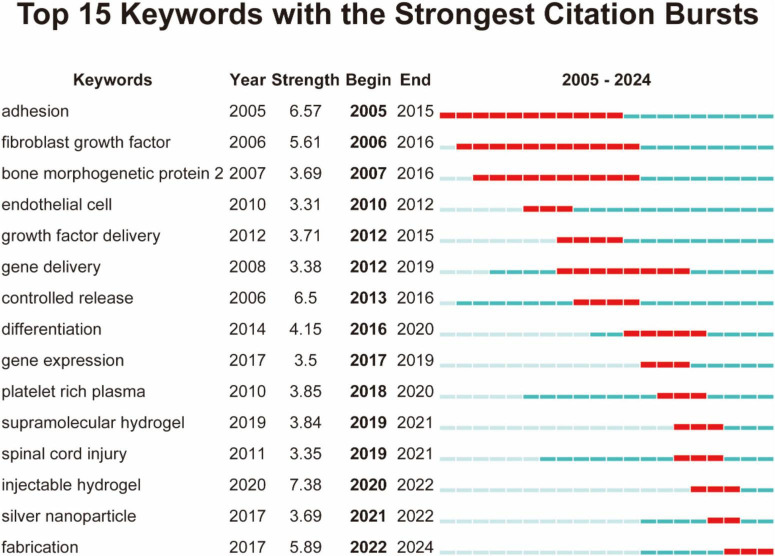
A detailed flowchart of the search and selection process, ensuring a systematic and comprehensive bibliometric evaluation. The initial search, applying restrictions on search terms and publication dates, yielded 912 publications. Further refinement based on publication type (Article and Review) and language (English) resulted in a final dataset of 885 publications selected for bibliometric analysis. This dataset spans seven key analytical dimensions: publication trends, countries/regions, institutions, keywords, references, research disciplines, and journals.

The retrieval strategy was defined as follows: Index = Science Citation Index Expanded (SCIE) of the Web of Science Core Collection (WOSCC); Search Terms = (T1: Biomaterials), (T2: Drug Delivery), (T3: Regenerative Medicine). The publication time span covered research published between January 1, 2005, and December 31, 2024. Only Article and Review document types were included, with the language restricted to English to ensure consistency in bibliometric analysis. Figure 9 provides a detailed overview of the complete retrieval and screening process, illustrating the methodology used to ensure a comprehensive and representative dataset.

### 5.3 Bibliometric analysis and visualization

Publication counts and citation data were directly retrieved from the Web of Science Core Collection (WOSCC). Data processing was conducted using GraphPad Prism 8.0, while the global distribution of publications was visualized through Microsoft PowerPoint 2021. This integrative approach effectively captured publication trends and the geographical spread of research over the 20-year period. To analyze national contributions, total publication output from leading countries was extracted and evaluated using WOSCC, R “bibliometrix,” and GraphPad Prism 8.0. Additionally, the H-index was calculated to assess the academic impact of individual researchers, providing a deeper understanding of publication influence and research leadership across different countries. VOSviewer was employed to construct and analyze co-citation, co-occurrence, and bibliographic coupling networks, facilitating a structured evaluation of research connectivity and thematic development. The global distribution of publications and inter-country collaboration patterns were then mapped using Microsoft PowerPoint 2021, further enhancing the visualization of research networks. To detect citation bursts, keyword trends, and reference clustering, CiteSpace (version 6.1) was utilized. This tool enabled the identification of key research trends and thematic clusters, offering valuable insights into the field’s evolution.

By integrating GraphPad Prism 8.0, VOSviewer, R “bibliometrix,” and CiteSpace, this study conducted a rigorous bibliometric analysis, focusing on publication output, citation patterns, and scholarly networks. The combination of these advanced analytical tools ensured a comprehensive, systematic, and high-resolution investigation of the research landscape in biomaterials-driven regenerative drug delivery.

## Data availability

The original contributions presented in the study are included in the article/supplementary material, further inquiries can be directed to the corresponding author.
